# Global Control and Regional Elimination of Measles, 2000–2012

**Published:** 2014-02-07

**Authors:** Robert T. Perry, Marta Gacic-Dobo, Alya Dabbagh, Mick N. Mulders, Peter M. Strebel, Jean-Marie Okwo-Bele, Paul A. Rota, James L. Goodson

**Affiliations:** 1 Department of Immunization, Vaccines, and Biologicals, World Health Organization, Geneva, Switzerland; 2 Division of Viral Diseases, National Center for Immunization and Respiratory Diseases; 3 Global Immunization Division, Center for Global Health, CDC

In 2010, the World Health Assembly established three milestones toward global measles eradication to be reached by 2015: 1) increase routine coverage with the first dose of measles-containing vaccine (MCV1) for children aged 1 year to ≥90% nationally and ≥80% in every district, 2) reduce and maintain annual measles incidence at <5 cases per million, and 3) reduce measles mortality by 95% from the 2000 estimate (1).* After the adoption by member states of the South-East Asia Region (SEAR) of the goal of measles elimination by 2020, elimination goals have been set by member states of all six World Health Organization (WHO) regions, and reaching measles elimination in four WHO regions by 2015 is an objective of the Global Vaccine Action Plan (GVAP).† This report updates the previous report for 2000–2011 (2) and describes progress toward global control and regional elimination of measles during 2000–2012. During this period, increases in routine MCV coverage, plus supplementary immunization activities (SIAs)§ reaching 145 million children in 2012, led to a 77% decrease worldwide in reported measles annual incidence, from 146 to 33 per million population, and a 78% decline in estimated annual measles deaths, from 562,400 to 122,000. Compared with a scenario of no vaccination, an estimated 13.8 million deaths were prevented by measles vaccination during 2000–2012. Achieving the 2015 targets and elimination goals will require countries and their partners to raise the visibility of measles elimination and make substantial and sustained additional investments in strengthening health systems.

## Immunization Activities

WHO and the United Nations Children’s Fund (UNICEF) use data from administrative records and surveys reported annually by member states to estimate MCV1 coverage among children aged 1 year.¶ Since 2003, member states also have reported the number of districts with ≥80% MCV1 coverage. Estimated MCV1 coverage increased globally from 73% to 84% during 2000–2009, then remained at 84% through 2012 (Table 1). The number of member states with ≥90% MCV1 coverage increased from 83 (43%) in 2000 to 128 (66%) in 2012. The number of member states with ≥90% MCV1 coverage nationally that also had ≥80% MCV1 coverage in all districts increased from 40 (38%) of 104 in 2003 to 58 (45%) of 128 in 2012. Of the estimated 21.2 million infants who did not receive MCV1 in 2012, approximately 13.5 million (64%) were in six member states: India (6.4 million), Nigeria (3.8 million), Ethiopia (1.0 million), Indonesia (0.9 million), Pakistan (0.7 million), and the Democratic Republic of the Congo (0.7 million).

During 2000–2012, the number of member states providing the second dose of measles vaccine (MCV2) through routine immunization services increased from 96 (50%) to 145 (75%). During 2012, approximately 145 million children received MCV during SIAs conducted in 33 member states. MCV coverage ≥95% after SIAs was reported by 18 (55%) member states, and 12 (36%) member states conducted coverage surveys to validate coverage. During measles SIAs, 20 (61%) member states included one or more additional child health interventions; 18 (55%) included oral poliovirus vaccination (Table 2).

## Disease Incidence

Effective measles surveillance includes case-based surveillance with laboratory testing to confirm cases. During 2004–2012,** the number of member states using case-based surveillance increased from 120 (62%) to 187 (96%).†† During 2000–2012, the number of member states with access to standardized quality-controlled testing through the WHO Measles and Rubella Laboratory Network increased from 71 (37%) to 191 (98%).§§

During 2000–2012, the number of measles cases reported worldwide each year¶¶ decreased 73%, from 853,480 to a historic low of 226,722, and measles incidence decreased 77%, from 146 to 33 cases per million population per year (Table 1). The decrease in 2012 occurred in all regions and followed 3 years of increasing numbers of cases. During 2000–2012, the Region of the Americas (AMR) maintained measles incidence at <5 cases per million; in 2012, reported incidence in the Western Pacific Region (WPR) was six cases per million, a historic low.

The percentage of reporting member states with <5 cases per million increased from 55% (104 of 188) in 2011 to 64% (119 of 187) in 2012. During 2012, large measles outbreaks were reported by the Democratic Republic of the Congo (72,029 cases), India (18,668), Indonesia (15,489), Ukraine (12,746), Somalia (9,983), Sudan (8,523), Pakistan (8,046), and Romania (7,450). China reported 6,183 cases, a historic low after a steady annual decrease from 38,159 cases in 2010.

Genotyping results from isolates from persons with measles were reported from 49 (39%) of the 125 member states reporting measles cases in 2012. Six measles genotypes were identified; the predominant genotypes were B3 in the African Region (AFR) and the Eastern Mediterranean Region (EMR); D4 in the European Region (EUR); H1, D8, and D9 in SEAR and WPR; with one G3 reported from one outbreak in WPR.***

## Mortality Estimates

In response to the lack of reliable data on the number of measles deaths from many member states, WHO has developed a model to estimate mortality using numbers and age distribution of reported cases, routine and SIA MCV coverage, and age-specific, country-specific case-fatality ratios (3,4). The model was refined in 2013 to reflect the impact of different SIA target age ranges and the population targeted in subnational SIAs. These refinements, together with new 2012 measles vaccination coverage and case data for all member states, updated data for the period before 2012 for some member states, and updated population estimates (5), led to new mortality estimates for 2000–2012. During this period, estimated measles deaths decreased 78%, from 562,400 to 122,000; all regions had substantial reductions in estimated measles mortality, ranging from 52% in EMR to 88% in AFR (Table 1). Compared with a scenario of no vaccination against measles, an estimated 13.8 million deaths were prevented by measles vaccination during 2000–2012 (Figure).

## Regional Verification of Measles Elimination

By 2012, regional verification commissions were established in AMR, EUR, and WPR, and frameworks for documenting elimination were developed in AMR and EUR. While verifying elimination, member states in AMR uncovered weaknesses in surveillance and routine immunization programs, leading to a regional emergency plan of action to strengthen these programs.

### Editorial Note

During 2000–2012, increasing routine MCV coverage worldwide and regular SIAs in member states lacking high coverage with 2 doses of MCV contributed to a 77% decrease in reported measles incidence and a 78% reduction in estimated measles mortality, reaching historic lows. During this period, measles vaccination prevented an estimated 13.8 million deaths. Measles elimination continues to be maintained in AMR (6), and WPR is approaching measles elimination (7). However, based on current trends and performance, the WHO Strategic Advisory Group of Experts (SAGE) concluded that the 2015 global targets and regional elimination targets in EUR, EMR, and AFR will not be achieved on time (8).

AFR, EMR, and SEAR, the regions with the largest number of infants not receiving MCV1 through routine immunization services in 2012, had large measles outbreaks during 2012 and had 98% of the estimated global measles mortality burden, highlighting the need to strengthen immunization systems. Globally 2012 might represent a temporary low in the normal cycle of measles incidence. Preventing a resurgence will require progress in reaching ≥95% of children with 2 MCV doses through routine immunization services and high-quality SIAs (9).

The findings in this report are subject to at least three limitations. First, MCV coverage estimates likely included errors resulting from inaccurate estimates of the size of target populations, inaccurate reporting of doses delivered, and inclusion of SIA doses given to children outside the target age group. Second, underestimation in surveillance data can occur because not all patients with measles seek care and not all of those who seek care are reported. These errors in coverage and surveillance data in turn affect the accuracy of the measles mortality model results. Finally, some member states also maintain multiple reporting systems for measles and might, like India, report aggregate, unconfirmed cases rather than case-based data.

To achieve measles elimination, member states should aim to fully implement measles control and elimination strategies described in GVAP and the 2012–2020 Global Measles and Rubella Strategic Plan (10) of the Measles and Rubella Initiative,††† which include achieving vaccination coverage ≥95% with 2 doses of MCV administered through routine immunization or SIAs and maintaining this coverage uniformly across all districts. For many member states now at <90% coverage nationally, reaching ≥95% coverage will require substantial and sustained additional investments of financial and human resources to strengthen health systems and achieve equitable access to immunization services. Further progress toward achieving the 2015 global measles control targets and regional measles elimination targets will also require member states and partners to increase the visibility of measles elimination activities and make the needed investments.

What is already known on this topic?

During 2000–2011, global vaccination coverage with the first dose of measles-containing vaccine increased from 72% to 84%, approximately 225 million children received a second opportunity for measles immunization during measles supplemental immunization activities in 2011, and global reported measles cases decreased until 2008, then increased in 2010 and 2011. By 2011, about 45% of countries had not met the incidence target of <5 cases per million. As milestones toward eventual global measles eradication, the 2010 World Health Assembly endorsed a series of targets to be met by 2015.

What is added by this report?

In 2012, estimated global coverage with the first dose of measles-containing vaccine remained at the 2011 level of 84%, but the number of countries providing a second dose of measles-containing vaccine through routine immunization services increased from 96 (50%) in 2000 to 145 (75%) in 2012, and 144 million children were vaccinated against measles during vaccination campaigns. In 2012, annual reported measles incidence was 33 reported cases per million population, a decline of 77% from 146 cases per million population in 2000, and estimated measles deaths decreased 78%, from 562,400 to 122,000. An estimated 13.8 million deaths were prevented by measles vaccination during 2000–2012.

What are the implications for public health practice?

Although measles incidence decreased during 2011–2012, the World Health Organization’s African, Eastern Mediterranean, and European regions are not on track to achieving their elimination targets. To accelerate progress toward achieving these regional measles elimination targets national governments and partners are urged to give these efforts high priority and adequate resources to achieve their commonly agreed upon goals, and in so doing reach the targets set by the Global Vaccine Action Plan.

## Figures and Tables

**FIGURE:**
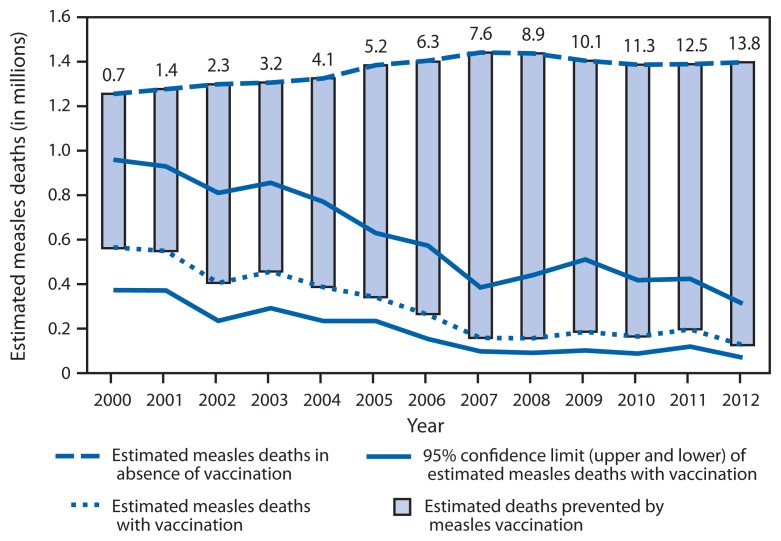
Estimated measles mortality and measles deaths prevented worldwide, 2000–2012* * Numbers over bars indicate cumulative estimated number of deaths prevented (in millions).

**TABLE 1:** Estimates of coverage with the first dose of measles-containing vaccine (MCV1) administered through routine immunization services among children aged 1 year, reported measles cases and incidence, and estimated measles mortality, by World Health Organization (WHO) region, 2000 and 2012

WHO region	2000						

	% coverage with MCV1*	% member states with coverage ≥90%	No. of reported measles cases†	Measles incidence (cases per million population)§¶	% member states with incidence <5 per million	Estimated measles deaths	

						No.	(95% CI)
African	53	9	520,102	841	8	354,900	(225,000–636,000)
Americas	93	63	1,755	2.1	89	<100	—
Eastern Mediterranean	72	57	38,592	90	17	53,900	(32,500–85,700)
European	91	60	37,421	50	48	300	(100–1,200)
South-East Asia	65	30	78,558	51	0	141,200	(105,800–186,400)
*South-East Asia (excluding India)*	*77*	*—*	*39,723*	*80*	*0*	*84,300*	*(67,800*–*103,200)*
India	59	—	38,835	37	0	56,900	(38,000–83,200)
Western Pacific	85	41	177,052	105	30	12,100	(6,800–48,500)
**Total**	**73**	**43**	**853,480**	**146**	**38**	**562,400**	**(370,200–957,900)**

WHO region	2012										

	% coverage with MCV1*	% member states with coverage ≥90%	No. of reported measles cases†	% decline from 2000	Measles incidence (cases per million population)§¶	% decline from 2000	% member states with incidence <5 per million	Estimated measles deaths		% mortality reduction 2000 to 2012	% total measles deaths in 2012

								No.	(95% CI)		
African	73	33	106,052	80	125	85	40	41,400	(13,900–148,500)	88	**34**
Americas	94	83	143	92	0.1	93	100	<100	—	—	**0**
Eastern Mediterranean	83	55	35,788	7	62	32	43	25,800	(17,500–42,200)	52	**21**
European	94	87	27,030	28	37	26	71	100	(0–1,300)	64	**0**
South-East Asia	78	55	46,945	40	26	50	36	52,700	(34,400–79,100)	63	**43**
*South-East Asia (excluding India)*	*88*	*—*	*28,277*	*29*	*47*	*41*	*40*	*36,200*	*(25,600–48,800)*	*57*	***30***
India	74	—	18,668	52	15	59	0	16,500	(8,800–30,300)	71	**14**
Western Pacific	97	74	10,764	94	6	94	70	2,000	(100–37,400)	84	**2**
**Total**	**84**	**66**	**226,722**	**73**	**33**	**77**	**64**	**122,000**	**(65,900–308,500)**	**78**	**100**

**Abbreviation:** CI = confidence interval.

* Based on WHO/UNICEF estimates of national immunization coverage, available at http://apps.who.int/immunization_monitoring/globalsummary/timeseries/tswucoveragemcv.html.

† Based on WHO reported measles case data, available at http://apps.who.int/immunization_monitoring/globalsummary/timeseries/tsincidencemeasles.html. Data for Region of the Americas available at http://ais.paho.org/phip/viz/im_vaccinepreventablediseases.asp.

§ Based on United Nations population data, available at http://esa.un.org/unpd/wpp/index.htm.

¶ Any country not reporting data on measles cases for that year was removed from both the numerator and denominator.

**TABLE 2:** Measles supplementary immunization activities (SIAs)* and the delivery of other child health interventions, by World Health Organization (WHO) region and member state, 2012

			Children reached in targeted age group		
				
WHO region / Member state	Age group targeted	Extent of SIA	No.	(%)†	Other interventions
**African**					
Burundi	6–59 mos	National	1,459,304	(103)	Vitamin A, anthelminthics
Cameroon	9–59 mos	National	3,570,032	(102)	Vitamin A
Chad	6–59 mos	National	2,270,772	(112)	OPV
Democratic Republic of the Congo	9–59 mos	Subnational	6,577,639	(102)	OPV
Eritrea	9–47 mos	National	277,928	(75)	OPV, vitamin A
Gabon	9–59 mos	National	168,749	(67)	Vitamin A, anthelminthics
Guinea	9–59 mos	National	2,098,829	(95)	OPV
Guinea Bissau	9–59 mos	National	220,263	(80)	Vitamin A, anthelminthics
Kenya	9–59 mos	National	5,995,049	(107)	OPV, vitamin A
Namibia	9 mos–14 yrs	National	885,259	(91)	OPV, vitamin A
Niger	9 mos–14 yrs	National	7,736,066	(102)	Vitamin A, anthelminthics
Sao Tome and Principe	9–59 mos	National	22,528	(105)	
Sierra Leone	9–59 mos	National	1,179,605	(102)	Vitamin A, anthelminthics
Uganda	9–59 mos	National	6,283,441	(100)	OPV, vitamin A, anthelminthics
Zambia	9 mos–14 yrs	National	7,503,515	(116)	OPV and tetanus toxoid vaccine, vitamin A
Zimbabwe	6–59 mos	National	1,613,437	(103)	OPV, vitamin A
**Americas**					
Haiti	9 mos–9yrs	National	2,963,911	(118)	OPV and rubella vaccine, vitamin A, anthelminthics
Honduras	1–4 yrs	National	696,712	(82)	OPV, mumps and rubella vaccines, vitamin A
Nicaragua	1–4 yrs	National	559,985	(107)	Rubella vaccine, vitamin A, anthelminthics
**Eastern Mediterranean**					
Afghanistan	9 mos–10 yrs	National	11,520,650	(103)	OPV
Djibouti	9–59 mos	National	96,064	(95)	
Iraq	9–60 mos	National	4,733,889	(94)	Rubella vaccine
Pakistan	9 mos–9 yrs	Rollover (national)§	1,954,175	(102)	OPV
Somalia	6–59 mos	Subnational children health days and SIAs in newly accessible areas	1,381,272	(90)	OPV and tetanus toxoid vaccine, vitamin A, anthelminthics
South Sudan	6– 59 mos	National	1,708,418	(90)	OPV, vitamin A
Syria	12–59 mos	National	768,086	(60)	Mumps and rubella vaccines
Yemen	6 mos–10 yrs	National	7,984,779	(93)	OPV, vitamin A
**South-East Asia**					
India	9 mos–10 yrs	Rollover (national)§	45,189,988	(84)	
Myanmar	9–59 mos	National	6,267,535	(97)	
Nepal	6 mos–14 yrs	National	9,685,099	(101)	Rubella vaccine
**Western Pacific**					
Mongolia	3–14 yrs	National	522,429	(93)	Rubella vaccine
Papua New Guinea	6–35 mos	National	552,872	(88)	OPV and tetanus toxoid vaccine, vitamin A, anthelminthics
Solomon Islands	12–59 mos	National	67,832	(101)	Rubella vaccine
**Total**			**144,516,112**		

**Abbreviation:** OPV = oral poliovirus vaccine.

* SIAs generally are carried out using two approaches. An initial nationwide catch-up SIA targets all children aged 9 months to 14 years; it has the goal of eliminating susceptibility to measles in the general population. Periodic follow-up SIAs then target all children born since the last SIA. Follow-up SIAs generally are conducted nationwide every 2–4 years and generally target children aged 9–59 months; their goal is to eliminate any measles susceptibility that has developed in recent birth cohorts and to protect children who did not respond to the first measles vaccination. The exact age range for follow-up SIAs depends on the age-specific incidence of measles, coverage with 1 dose of measles-containing vaccine, and the time since the last SIA.

† Values >100% indicate that the intervention reached more persons than the estimated target population.

§ Rollover national campaigns started the previous year or will continue into the next year.

## Footnotes

* Whereas the coverage milestone is to be met by every member state, the incidence and mortality reduction milestones are to be met globally.

† The Decade of Vaccines is a collaboration between WHO, UNICEF, the Bill and Melinda Gates Foundation, the GAVI Alliance (formerly the Global Alliance for Vaccines and Immunization), the U.S. National Institute of Allergy and Infectious Diseases, the African Leaders Malaria Alliance, and others to extend, by 2020 and beyond, the full benefit of immunization to all persons. Additional information available at http://apps.who.int/gb/ebwha/pdf_files/wha65/a65_22-en.pdf.

§ SIAs generally are carried out using two target age ranges. An initial, nationwide catch-up SIA targets all children aged 9 months–14 years, with the goal of eliminating susceptibility to measles in the general population. Periodic follow-up SIAs then target all children born since the last SIA. Follow-up SIAs generally are conducted nationwide every 2–4 years and target children aged 9–59 months; their goal is to eliminate any measles susceptibility that has developed in recent birth cohorts and to protect children who did not respond to the first measles vaccination.

¶ Among children aged 1 year or, if MCV1 is given at age ≥1 year, among children aged 24 months. WHO/UNICEF estimates of national immunization coverage are available at http://www.who.int/immunization_monitoring/routine/immunization_coverage/en/index4.htm.

** Data for years before 2004 were not available.

†† Member states without case-based measles surveillance in 2012 include Djibouti, India, Mauritius, Seychelles, Sao Tome and Principe, Somalia, and South Sudan.

§§ Member states without access to standardized quality-controlled testing by the WHO Measles and Rubella Laboratory Network in 2012 included Cape Verde, Sao Tome and Principe, and Seychelles.

¶¶ Data available at http://apps.who.int/immunization_monitoring/globalsummary/timeseries/tsincidencemeasles.html.

*** Data available from the Measles Nucleotide Surveillance (MeaNS) database at http://www.hpa-bioinformatics.org.uk/Measles/Public/Web_Front/main.php.

††† The Measles and Rubella Initiative is a partnership established in 2001 as the Measles Initiative, led by the American Red Cross, CDC, the United Nations Foundation, UNICEF, and WHO. Additional information available at http://www.measlesrubellainitiative.org.
